# Publisher Correction: Fungal antigenic variation using mosaicism and reassortment of subtelomeric genes’ repertoires

**DOI:** 10.1038/s41467-023-43993-7

**Published:** 2023-12-01

**Authors:** Caroline S. Meier, Marco Pagni, Sophie Richard, Konrad Mühlethaler, João M. G. C. F. Almeida, Gilles Nevez, Melanie T. Cushion, Enrique J. Calderón, Philippe M. Hauser

**Affiliations:** 1 Institute of Microbiology, Lausanne University Hospital and University of Lausanne, Lausanne, Switzerland; 2 Vital-IT Group, SIB Swiss Institute of Bioinformatics, Lausanne, Switzerland; 3 Institute for Infectious Diseases, University of Bern, Bern, Switzerland; 4 UCIBIO, Applied Molecular Biosciences Unit, Department of Life Sciences, NOVA School of Science and Technology, Universidade NOVA de Lisboa, 2829−516 Caparica, Portugal; 5 Laboratoire de Parasitologie et Mycologie, Hôpital de La Cavale Blanche, CHU de Brest, Brest, France; 6 Infections respiratoires fongiques (IFR), Université d’Angers, Université de Brest, Brest, France; 7 Department of Internal Medicine, Division of Infectious Diseases, College of Medicine, University of Cincinnati, Cincinnati, OH 45267 USA; 8 Cincinnati VAMC, Medical Research Service, Cincinnati, OH 45220 USA; 9 Instituto de Biomedicina de Sevilla, Hospital Universitario Virgen del Rocίo/Consejo Superior de Investigaciones Cientίficas/Universidad de Sevilla, Seville, Spain; 10 Centro de Investigación Biomédica en Red de Epidemiologίa y Salud Pública, Servicio de Medicina Interna, Hospital Universitario Virgen del Rocίo, Departamento de Medicina, Facultad de Medicina, Seville, Spain

**Keywords:** Fungal immune evasion, Fungal genetics

Correction to: *Nature Communications* 10.1038/s41467-023-42685-6, published online 02 November 2023

The original version of this Article contained an error in Fig. 1. In panels a and b the 3 black stars in each panel identifying the patients infected by a single strain were missing.  These have now been added in panel a and b in Fig 1.

In addition, in the Result section of the main text the tabs labelled “Table 1” and “Table 2” in Supplementary Data 6 were incorrectly referred to as “Supplementary Table 1” and Supplementary Table 2”; these are now referred to as “Table 1 in Supplementary Data 6” in the first and fourth sentence of the fifth paragraph, and “Table 2 in Supplementary Data 6” in the fifth sentence of the seventh paragraph.

These errors have been corrected in both the PDF and HTML versions of the article.

