# Supplementary material for: Fungal antigenic variation using mosaicism and reassortment of subtelomeric genes’ repertoires
Source: Nat Commun. 2023 Nov 2;14:7026. doi: 10.1038/s41467-023-42685-6 (PMC10622565; doi:10.1038/s41467-023-42685-6)
Supplement: Supplementary file 1 — Supplementary Information [file 41467_2023_42685_MOESM1_ESM.pdf]

Fungal antigenic variation using mosaicism and reassortment of subtelomeric genes' repertoires

Meier et al.

Supplementary information

Table of contents :

Supplementary notes **1**

1. Reproducibility of the determination of the repertoires 1
2. Multitarget genotyping to assess infection by a single *P. jirovecii* strain 2
3. Confirmation of the allele abundance using amplicon subcloning 3
4. Search of duplicated fragments within the subtelomeres of a single strain 3
5. Specificity of the CRJE sequence for *P. jirovecii* 5
6. Absence of site-specific recombinase targeting the CRJE sequence 5
7. Comparison of the CRJE sequences of different *Pneumocystis* species 6

Supplementary tables **8**

Supplementary figures **18**

Supplementary References **40**

Supplementary notes

1. Reproducibility of the determination of the repertoires

In order to investigate the reproducibility of the whole methodology, eight samples were analysed twice in independent experiments using identical conditions and protocol, except for the DNA extraction that was performed only once. The alleles identified and their abundance were highly similar in the duplicates of four genomic repertoires with 73 to 98% of alleles in common (Supplementary table 6, Supplementary Fig. 4a). Moreover, all alleles identified in only one of the duplicates were low abundant (less than 1% of all reads composing the repertoire, gray and light

green lines in Supplementary Fig. 4a). These alleles were presumably not amplified in one duplicate. This might result from a varying efficiency of amplification and/or PacBio sequencing of the alleles, some being not amplifiable at all (see Results). Thus, the number of alleles in each repertoire might be underestimated to an unknown extent. Moreover, in the case of the expressed repertoires, the efficiency of amplification has probably been decreased by the fact that the forward primer GSK135 within the UCS matches multiple regions of the human genome.

As far as the expressed repertoires are concerned, there were more differences between the duplicates than for the genomic repertoires with only 33 to 53% of alleles in common. For two samples (BE1 and LA1), most alleles (97 to 100%) that were not present in both duplicates had an abundance lower than 1%, as observed for the genomic repertoires (Supplementary table 6). On the other hand, the values for samples LA3 and LA4 were only 33 and 50%, respectively. This might result from the lower number of alleles in these two latter samples (14 to 22 versus 82 to 140 in BE1 and LA1). The reduced reproducibility for the expressed repertoires might be due to the greater variation in the allele abundances than in the genomic repertoires (see Results, section "Abundance of the *msg*-I alleles in the patients"), a phenomenon that could be increased when the number of alleles present decreases. Moreover, the varying efficiency of amplification and/or PacBio sequencing of some alleles may also increase the phenomenon.

2. Multitarget genotyping to assess infection by a single *P. jirovecii* strain

We further investigated the number of *P. jirovecii* strains present in the nine patients infected by a single strain according to the PacBio ITS1-5.8-ITS2 genotyping. We used the genotyping method consisting in subcloning PCR products of four markers followed

by Sanger sequencing of subclones to detect co-infections [18,19]. The presence of two alleles of at least one marker for six of the nine patients revealed the presence of at least one supplementary co-infecting strain (Supplementary table 7).

3. Confirmation of the allele abundance using amplicon subcloning

The last step of the bioinformatics pipeline provided the abundance of each allele in each sample in percentage of all reads composing the repertoire. A wet-lab approach was used to verify these abundance values. Amplicons from the expressed repertoire of patients LA7, BR3 and SE3 used for PacBio CCS were subcloned using the TOPO cloning kit (Invitrogen). These samples were selected because the low numbers of alleles composing their expressed repertoires facilitates the estimation of abundances by subcloning. Despite the low accuracy of the subcloning approach due to the small number of subclones analysed (8 to 19), the abundances obtained are consistent with those observed using Pacbio CCS followed by the bioinformatics pipeline (Supplementary table 8).

4. Search of duplicated fragments within the subtelomeres of a single strain

We searched for all duplicated fragments of size ≥ 100 bps present within the 10 representative subtelomeres assembled from a single strain (Supplementary Fig. 7). We used BLASTn comparisons of the 35 genes, 15 pseudogenes, and 50 intergenic spaces covering integrally these subtelomeres to the *P. jirovecii* PacBio genome assembly. The significant hits obtained for each gene corresponded to genes and pseudogenes of the same family, whereas pseudogenes generated few hits. The hits obtained for the intergenic spaces were intergenic spaces of the subtelomeres, which were upstream of genes or pseudogenes of the same family as for the query. However,

families II and III constituted an exception because some of their upstream sequences produced reciprocal hits with a low score or coverage of the query, which was consistent with the observations made in the section “Sequences flanking the *msg* genes” of the Results. Visual inspection of all alignments of the queries with their hits identified 61 and 38 duplicated fragments involving respectively 14 genes, 5 pseudogenes and 17 intergenic spaces, with a length up to 1142 bps (Supplementary table 9). We did not detect any duplicated fragments between the genes and intergenic spaces of family VI, nor between those of families II and III. Alignments of a number of mosaic genes, for example *msg*-I no. 45 and 94 (Supplementary Fig. 11), suggested that the regions between the shared fragments presented a sequence identity close to those observed on average between the members of each *msg* family (66 to 83%) [1]. The presence of at least one of these duplicated fragments suggests that 22 to 100% of genes, pseudogenes, or intergenic spaces of families I to V are mosaic. These proportions are in fair agreement with those we previously observed [1] for genes and pseudogenes, including the absence of mosaicism in family VI (Supplementary table 9). Five out of the 15 pseudogenes investigated were also concerned by the phenomenon. These observations confirm the mosaicism of *msg* genes and pseudogenes and reveal that the intergenic spaces are also concerned.

The 99 duplicated fragments detected are represented on the 10 representative subtelomeres that we analysed (Supplementary Fig. 7), as well on the 27 supplementary subtelomeres (Supplementary Fig. 8). Inspection of Supplementary Fig. 7 revealed the following features:

(i) Most subtelomeres share fragments with many other subtelomeres.

(ii) Some genes and their upstream space presented many duplicated fragments (for example, gene *msg*-II no.13 in subtelomere 26, and *msg*-III no. 53 in subtelomere 74). Sequence alignments revealed that these fragments sometimes overlap or are identical. This is also revealed by the locations of the shared fragments within the query (Supplementary Data 3 and 4). The important variation of the latter locations suggest that no hotspots of recombination are not present along the *msg* genes.

(iii) The entirety of the partial *msg*-I gene no. 52 at the end of subtelomere 74 (Supplementary Fig. 7) is duplicated in subtelomere 95 (subtelomere/contig 95, gene no. 61, Supplementary Fig. 8b), suggesting a whole gene duplication.

5. Specificity of the CRJE sequence for *P. jirovecii*

Because of the peculiarity of the CRJE, we wondered if it was specific to *P. jirovecii* by using it as query in a BLASTn analysis against the whole nucleotide collection (nr/nt). The full CRJE sequence of 33 bps is present only in *P. jirovecii*, and only at the beginning of *msg*-I genes. Nevertheless, the 25 bps sequence covering the mirror repeats, *i.e.* positions 2 to 26 in Fig. 8a, was present in few copies and few other organisms (2 copies in 1 bacterial species, and 1 to 4 copies in 7 different butterfly species).

6. Absence of site-specific recombinase targeting the CRJE sequence

To investigate if of a recombinase that recognizes specifically the CRJE sequence exists in *P. jirovecii*, we performed extensive homology searches using BLASTn involving site-specific recombinases as bait from various organisms (see

Methods). Indeed, the CRJE includes a mirror repeat and such enzymes recognize generally repeats[17]. These analyses did not detect any recombinases of interest.

7. Comparison of the CRJE sequences of different *Pneumocystis* species

We investigated if the CRJE sequence of other species than *P. jirovecii* can also potentially form *H-DNA triplex and encode several R residues. Eleven CRJEs are shown in Supplementary Fig. 13a and the alignments of their DNA sequences and encoded peptides are shown with their corresponding phylogenic trees in Supplementary Fig. 13b. The trees are consistent with those previously reported on the basis on the entire UCS including the CRJE[20] or on 106 single-copy genes[21] (in the latter one, *P. wakefieldiae* is close to *P. carinii* and *P. murina* possibly because of the absence of *P.* sp. *"exulans"* and *P.* sp. *"tanezumi"*). These trees reveal three groups of CRJEs and one outlier.

Group 1 includes CRJEs of two *Pneumocystis* species specific to primates and one to rabbit (*P. jirovecii, P. macacae, P. oryctolagi*). Although that of *P. oryctolagi* is less symmetrical, the three CRJE sequences might potentially form *H-DNA because they closely resemble the canonical mirror repeat reported to do so (see Results, section "Structure of the CRJE sequence present at the beginning of each *msg*-I gene"). The peptide encoded by the CRJE of *P. jirovecii* presents a repetition of the motif ARAV that is not observed in those of *P. macacae* and *P. oryctolagi*. However, the two latter present also two R residues in addition to that present in the Kexin recognition site KR that might be recognized by a further protease. This might further ensure the proper removal of the constant part of the Msg-I proteins that is believed to be carried out by the Kexin at the site KR (see Results). Interestingly, these two R residues are encoded by a codon including bases that are imperfect in the mirror repeat, as in *P. jirovecii*.

Group 2 includes CRJEs of three *Pneumocystis* species specific to different rat species and one to mouse (*P. carinii, P. murina, P.* sp*. "fluvescens", P.* sp*. "muelleri"*). These CRJEs are less likely to form *H-DNA triplex because they present less symmetry and no clear mirror repeat as compared to the canonical sequence. Nevertheless, the strand shown is enriched in purines suggesting that formation of triplex might occur. Indeed, the formation of *H-DNA triplex is more versatile and less requiring at the level of sequence than canonical H-DNA [22]. Out of the four CRJEs of this group, only that of *P. carinii* presents a supplementary R residue in the encoded peptide in addition to the KR site.

Group 3 includes CRJEs of three *Pneumocystis* species specific to different rat species (*P. wakefieldiae, P.* sp*. "tanezumi", P.* sp*. "exulans"*). These CRJEs are more distant from the canonical mirror repeat than those of group 2, suggesting that formation of *H-DNA triplex by them is even less likely. Nevertheless, they present each a stretch enriched in purines that may form a triplex according to Mirkin [22]. They do not present supplementary R residues in their encoded peptide.

The outlier CRJE of *P. canis* encodes one supplementary R residue.

Supplementary tables

Supplementary Table 1. BAL samples from 24 immunocompromised patients analysed in this study.

City	Country	Patient code [a]	Collection year	Underlying disease	Number of *P. jirovecii* genomes / ml
Lausanne	Switzerland	LA1	2014	HIV	8.94E+07
		LA2	2014	HIV	6.84E+07
		LA3	2014	unknown	5.74E+07
		LA4	2018	unknown	5.33E+06
		LA5	2014	unknown	3.11E+06
		LA6	2017	unknown	1.11E+09
		LA7	2014	HIV	6.10E+07
		LA8	2012	HIV	2.29E+07
		LA9	2014	HIV	3.66E+06
Bern	Switzerland	BE1	2014	HIV	2.33E+08
		BE2	2013	kidney transplant	1.81E+07
		BE4	2015	cancer	1.27E+07
		BE5	2014	cancer	4.24E+06
Brest	France	BR1	2018	giant cell arteritis	1.98E+06
		BR2	2018	cancer	1.02E+06
		BR3	2019	HIV	6.89E+07
		BR4	2020	psoriasis (methotrexate)	3.82E+06
		BR5	2019	cancer	1.42E+07
Cincinnati	US	CI1	2009	unknown	ND [b]
		CI5	1995	unknown	ND
Seville	Spain	SE1	2007	HIV	1.65E+06
		SE2	2010	HIV	6.39E+08
		SE3	2013	HIV	1.15E+06
		SE4	2013	HIV	8.47E+06

[a] LA, Lausanne. BE, Bern. BR, Brest. CI, Cincinnati. SE, Seville.

[b] ND, not determined.

Supplementary Table 2. R packages used with the R version 4.1.0 (2021-05-18).

R package	Version	Description	Reference
BiocManager	1.30.16	access bioconductor package repository	2
biostrings	2.62.0	manipulation of biological strings	3
DECIPHER	2.22.0	manage biological sequences	4
dendextend	1.15.2	dendogram manipulation	5
ggplot2	3.3.5	figures and plots	6
gplots	3.1.1	create heatmaps	7
gridExtra	2.3	arrange plots	8
pBrackets	1.0.1	bracket elements in plot	9
plotrix	3.8-2	plot options	10
reshape2	1.4.4	reshape data	11
seqinr	4.2-8	manipulation of sequences	12
stringr	1.4.0	string operations	13

Supplementary Table 3. Primers used for the control PCRs specific to given alleles.

Allele	Name allele PacBio	Present in patients [a]	Primer	Position (nt)	Sequence (5' - 3')
A	CI3c106432837	LA2, LA8, BE1, CI3	A-for	198-224	CCAAGTTGTAAAGGTAGTAAATGTAGC
			A-rev	1909-1929	TAAACGACTTCGTGTCTTTTG
B	LA2cd106037614	LA2, CI3	B-for	52-70	GTCCACCTCTAGTGCAAGC
			B-rev	1627-1651	TTCTTGACATTTCCATTTTTATTAG
C	LA7c108528291	LA2, BE1, CI3	C-for	57-80	GAAAAGACATGTGAGAACCTTATG
			C-rev	1791-1812	GCTTTTCTAGTTCTTTTCTTGC

[a] BE, Bern. CI, Cincinnati. LA, Lausanne.

Supplementary Table 4. Genotypes identified among the 24 patients of this study.

PacBio ITS1-5.8S-ITS2 genotype	Genotype number of Xue et al. (2019)[23]	GenBank accession number	Number observations among the 24 patients
Pj01	1	JQ365709	12
Pj02	17	AB481410	11
Pj03	59	MK300661	5
Pj04	10	JQ365725	2
Pj05	ND [a]	JQ365722	3
Pj06	12	AB481406	4
Pj07	62	MK300664	2
Pj08	27	JQ365707	2
Pj09	ND	OR475686 [b]	1
Pj10	4	KC470776	2
Pj12	ND	OR475687 [b]	1
Pj14	ND	OR475688 [b]	1
Pj15	ND	OR475689 [b]	1
Pj16	ND	OR475690 [b]	1
Pj17	ND	OR475691 [b]	1
Pj18	9	JQ365723	1
Pj19	ND	OR475692 [b]	1
Pj20	ND	OR475693 [b]	1
Pj21	ND	OR475694 [b]	1
Pj22	ND	OR475695 [b]	1
Pj24	ND	OR475696 [b]	1
Pj25	ND	OR475697 [b]	1
Pj26	ND	OR475698 [b]	1
Pj27	ND	OR475699 [b]	1
Pj28	ND	OR475700 [b]	1

[a] ND, not described.
[b] This study.

Supplementary Table 5. Characteristics of the expressed and genomic *msg*-I gene repertoires observed in the 24 patients.

Patient [a]	Number of alleles in		Number of alleles in common between the two repertoires	% expressed in genomic (alleles in common / total number in expressed)	% genomic in expressed (alleles in common / total number in genomic)	Number of *P. jirovecii* strains	PacBio ITS1-5.8S-ITS2 Pj genotype numbers [b]
	expressed repertoire	genomic repertoire					
LA1	82	148	72	88	49	3	2,6,14
LA2	54	49	44	81	90	1	4
LA3	22	79	20	91	25	1	1
LA4	18	130	14	78	11	3	4,6,18
LA5	21	144	19	90	13	2	1,10
LA6	91	116	82	90	71	5	2,17,20,21,26
LA7	5	59	5	100	8	1	3
LA8	59	105	51	86	49	1	1
LA9	7	54	7	100	13	1	1
BE1	90	185	59	66	32	4	1,3,5,8
BE2	63	56	56	89	100	1	1
BE4	82	113	79	96	70	3	1,2,27
BE5	18	118	9	50	8	5	2,5,6,8,28
BR1	3	83	3	100	4	3	1,2,24
BR2	2	96	2	100	2	3	1,3,19
BR3	5	128	2	40	2	3	2,5,25
BR4	9	96	5	56	5	2	3,15
BR5	18	170	17	94	10	4	2,3,6,10
CI1	108	140	100	93	71	4	1,2,7,12
CI5	45	44	32	71	73	1	2
SE1	41	61	37	90	61	1	1
SE2	12	115	10	83	9	1	1
SE3	8	77	8	100	10	3	2,7,9
SE4	17	139	17	100	12	3	2,16,22

[a] LA, Lausanne. BE, Bern. BR, Brest. CI, Cincinnati. SE, Seville.

[b] The genotypes are described in Supplementary table 4.

Supplementary Table 6. Duplicated analyses of eight samples.

Repertoire	Patient [a]	Number alleles			Common alleles in duplicates		% of different alleles with abundance <1%
		duplicate 1	duplicate 2	Total distinct	number	% (alleles in common / total number distinct alleles)	
genomic	LA2	49	48	49	48	98	100
	LA1	149	134	154	129	84	100
	BE1	185	211	212	184	87	100
	LA3	79	80	92	67	73	100
expressed	BE1	90	121	138	73	53	100
	LA1	82	140	148	74	50	97
	LA3	22	19	28	13	46	33
	LA4	18	14	24	8	33	50

[a] LA, Lausanne. BE, Bern.

Supplementary Table 7. Alleles detected using multitarget genotyping of *P. jirovecii* [a].

Patient	ITS1 [b]	26S	mt26S	β-tub
LA2	B (13)	1 (14)	8 (16)	1 (15)
LA3	B (12)	1 (1), 4 (4)	8 (5)	3 (5)
LA7	A2 (5)	4 (6)	1 (1), 2 (4)	3 (5)
LA8	B (19)	4 (1), 6 (4)	4 (1), 6 (4)	3 (9)
LA9	B (19), B2 (5)	-	-	-
BE2	B (13)	1 (13)	3 (14)	1 (13)
CI5	B (15)	1 (15)	7 (15)	3 (15)
SE1	B (5)	1 (1), 4 (5)	3 (5)	3 (5)
SE2	B (5)	4 (6)	3 (3), 7 (2)	3 (6)

[a] The nine patients supposedly infected by a single strain according to PacBio ITS1-5.8-ITS2 genotyping were analysed. The numbers or letters of the alleles used in reference [18] are given, except for the 26S allele 6 that was described only in reference [19]. The 26S allele 4 is a new allele identical to allele 2 except the presence of a A at nucleotide position 3. The numbers in parentheses are those of the clones with the given allele. ITS1, internal transcribed spacer number 1 of the nuclear rRNA genes operon. 26S, intron of the nuclear 26S rRNA gene. mt26S, variable region of the mitochondrial 26S rRNA gene. β-tub, region surrounding intron number 6 of the β-tubulin gene.

[b] The ITS1 sequences observed were identical to those observed by PacBio sequencing (Supplementary table 5), except sample LA9 that harboured in addition allele B2.

Supplementary Table 8. Abundance of alleles determined using PacBio CCS and subcloning.

			Subcloning	
Patient [a]	Allele name [b]	Abundance PacBio (%)	Abundance (%)	Nb of clones
BR3	BR3u100403135	33	74	14
	BR3u100532918	32	0	0
	BR3u103089526	24	21	4
	BR3u100992345	10	5	1
	BR3u119802438	2	0	0
LA7	LA7u47056848	72	60	6
	LA7u100272069	9	0	0
	LA7u10029856	8	10	1
	LA7u101910862	7	20	2
	LA7u100074959	4	10	1
SE3	SE3u100009239	35	38	3
	SE3u100271002	14	13	1
	BR1u102369376 [b]	13	13	1
	SE3u100008413	11	13	1
	CI1u100600372 [b]	9	0	0
	SE3u117048602	8	25	2
	SE3u105186188	7	0	0
	SE3u110953735	4	0	0

[a] LA, Lausanne. BR, Brest. CI, Cincinnati. SE, Seville.

[b] The name of these alleles results from their high abundance in samples BR1 or CI1.

Supplementary Table 9. Duplicated fragments ≥ 100 bps within the10 *P. jirovecii* representative subtelomeres from a single strain [a].

	msg family	No. of genes or intergenic space upstream of genes (of which pseudogenes): used as query	No. of genes or intergenic space upstream of genes (of which pseudogenes): with duplicated fragments ≥ 100 bps	Total no. of duplicated fragments ≥ 100 bps detected [b] (of which between genes and pseudogenes)	Mean size (bps) of duplicated fragments (range)	% potential mosaic genes or intergenic space (pseudogenes included)	% potential mosaic genes in reference 1[c]
Genes and pseudogenes	I	20 (8)	5 (2)	7 (2)	513 (109-670+1142) [d]	25	42
	II	9 (3)	5 (0)	32 (0)	249 (117-710)	56	28
	III	5 (0)	3 (0)	12 (0)	120 (100-153)	60	40
	IV	2 (2)	2 (2)	4 (4)	165 (113-221)	100	22
	V	7 (1)	4 (1)	7 (1)	183 (102-363)	57	7
	VI	4 (0)	0 (0)	0 (0)	0	0	0
	outlier	3 (1)	0 (0)	0 (0)	0	0	-
	**Total**	**50** (15)	**19** (5)	**61** (7)			
Intergenic spaces	I	20 (8)	9 (1)	12 (1)	148 (105-224)	45	-
	II	9 (3)	2 (0)	6 (0)	314 (131-787)	22	-
	III	5 (0)	2 (0)	6 (0)	233 (132-320)	40	-
	IV	2 (2)	1 (1)	2 (2)	129 (115, 142)	50	-
	V	7 (1)	3 (1)	12 (3)	178 (115-334)	43	-
	VI	4 (0)	0 (0)	0 (0)	0	0	-
	outlier	3 (1)	0 (0)	0 (0)	0	0	-
	**Total**	**50** (15)	**17** (3)	**38** (6)			

Footnotes of Supplementary Table 9:

[a] All *msg* genes, pseudogenes, and intergenic spaces composing the 10 representative subtelomeres shown in Supplementary Fig. 7 were used as query in BLASTn analyses (search of somewhat similar sequences) against the PacBio *P. jirovecii* genome assembly [1]. Each upstream intergenic space extended up to the end of the gene located upstream (all genes are oriented towards the telomere within the subtelomeres, see Supplementary Fig. 7). Visual inspection of all alignments of the query with the significant hits identified the duplicated fragments. Outlier genes are those that could not be attributed to one of the six *msg* families [1].

[b] The duplicated fragments detected twice because of reciprocal BLASTn analyses were counted only once.

[c] Using various numerical bioinformatics tools to detect recombination events between *msg* genes and pseudogenes [1].

[d] The duplicated fragment of 1142 bps corresponds to the entirety of the partial gene *msg*-I no. 52 at the end of contig 74 (Supplementary Fig. 7).

Supplementary figures

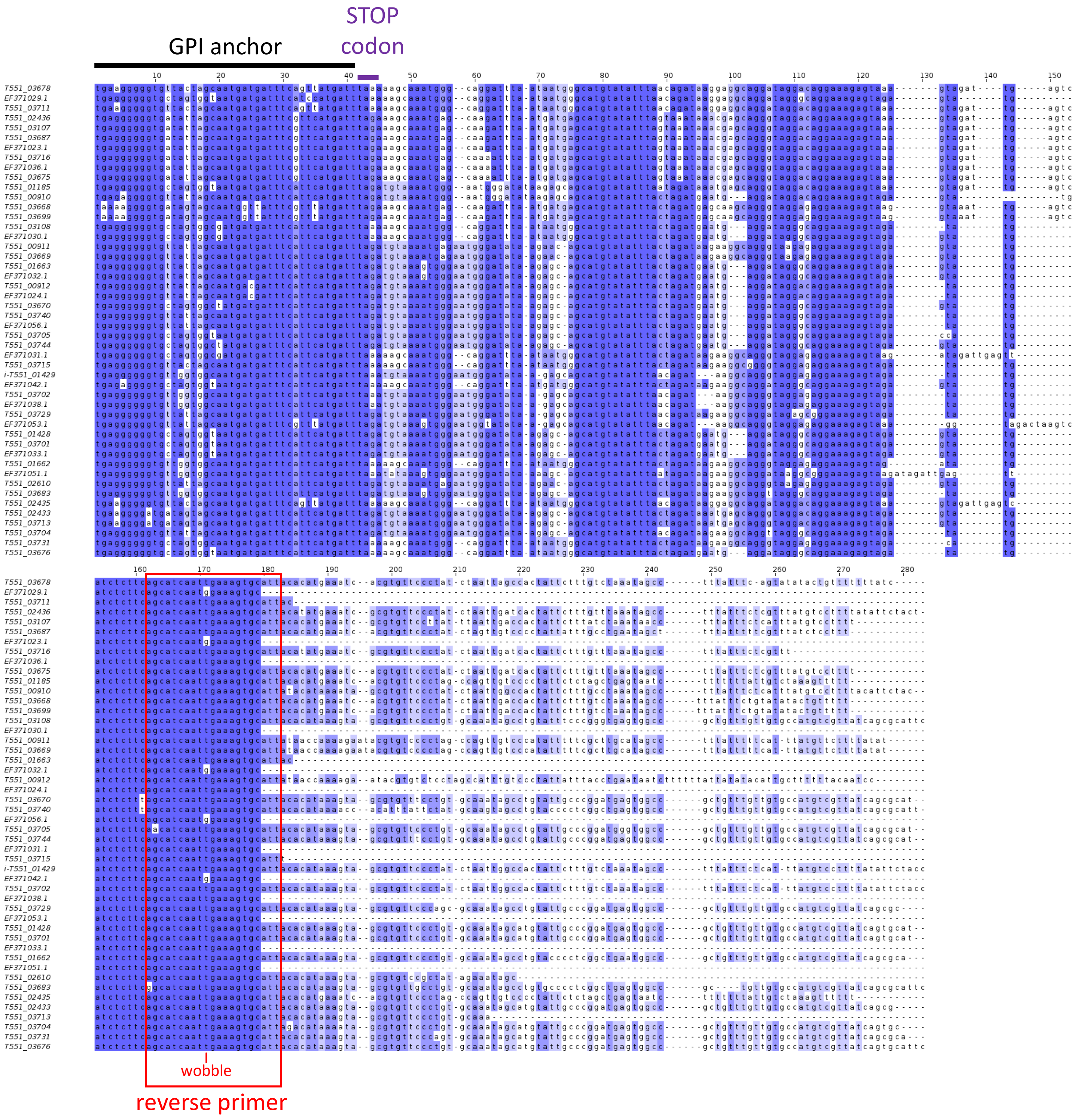

Supplementary Figure 1. Alignment of the 48 published *msg*-I gene sequences that include the conserved region of 31 bps ca. 90 bps after the stop codon. The gene IDs of the sequences and the localization of the reverse primer used to amplify both the genomic and expressed repertoires are indicated.

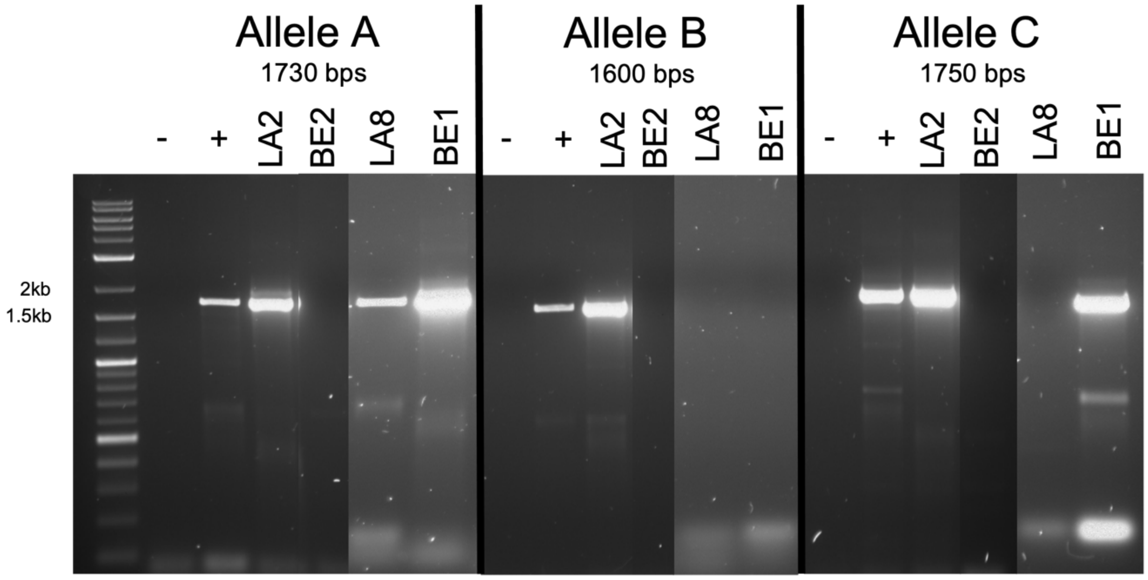

Supplementary Figure 2. **Analysis in agarose gel of the PCRs using primers specific to fragments of *msg*-I alleles A, B and C.** The size of the PCR product from each allele is indicated. The positive control (+) is randomly amplified DNA of patient LA2 that contains all three alleles. The primers used and alleles are described in Supplementary Table 3. Data are provided in the Source Data file.

10xT
GAAAATTCAGCTTAAACACTTCCCTAGTGTTTTAGCATTTTTCAAACATCTGTGAATTTTTT

6xT
TTTTGTTTGGCGAGGAGCTGGCTTTTTTGCTTGCCTCGCCAAAGGTGTTTATTTTTAAAATT

TTAAATTGAATTTCAGTTTTAGAATTTTTTAAAAACTTTCAACAATGGATCTCTTGGCTCTC

GCGTCGATGAAGAACGTGGCAAAATGCGATAAGTAGTGTGAATTGCAGAATTTAGTGAATCA

TCGAATTTTTGAACGCATCTTGCGCTCCTTAGTATTCTAGGGAGCATGCCTGTTTGAGCGTT

5xT 5xA 3xA
ATTTTTAAGTTCCTTTTTTCAAGCAGAAAAAAGGGGATTGGGCTTTGCAAATATAATTAGAA

TAAAATAATTATATGCATGCTAGTCTGAAATTCAAAAGTAGCTTTTTTTCTTTGCCTAGTGT

4xT
CGTAAAAATTCGCTGGGAAAGAAGGAAAAAAGCTTTTATAAATACAAGAATT

Supplementary Figure 3. ***P. jirovecii* ITS1-5.8S-ITS2 sequence (JQ365709.1)**. Highlighted in yellow are the six homopolymers that were homogenized in the present study, *i.e.* they were replaced in all sequences by the number of nucleotides indicated in red.

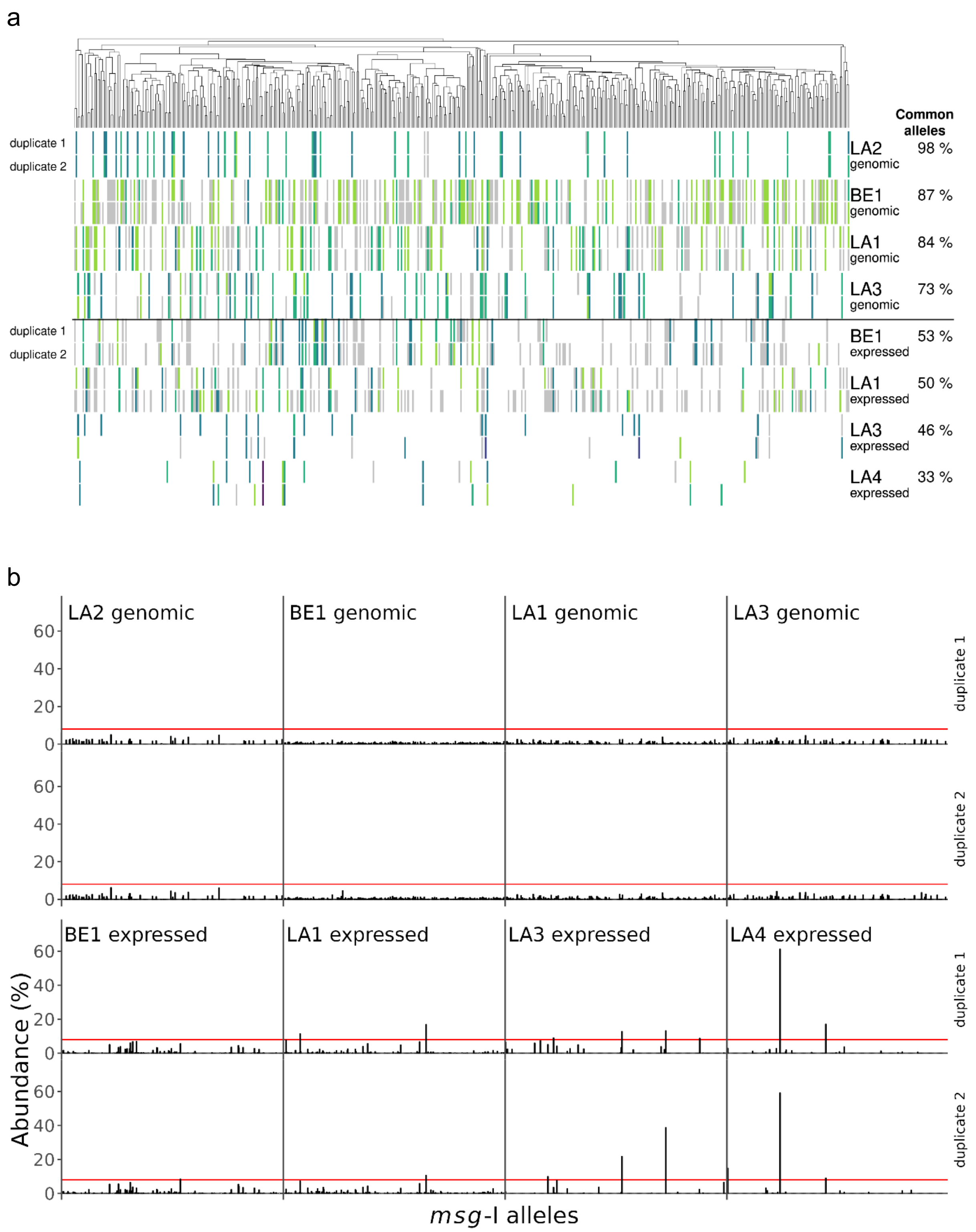

Supplementary Figure 4. Duplicate analyses to evaluate the reproducibility of the whole methodology. The bottom duplicate of each sample is also shown in Figures 1a and 1b. Patient LA2 was infected by a single strain. LA, Lausanne. BE, Bern. Data are provided in the Source Data file.

a. Composition of the genomic and expressed *msg*-I repertoires observed. Each vertical line of the heatmap represents an allele present in the given repertoire with the color figuring its abundance in % of all reads composing the repertoire, as indicated at the top right of the figure. The 470 alleles observed were sorted using hierarchical classification trees of the multiple alignments of the allele sequences (Fitch distance, average linkage). The percentage of common alleles between the duplicates are indicated next to the patient's name.

b. Abundance of the alleles within the genomic and expressed repertoires (see Results, section "Abundance of the *msg*-I alleles in the patients"). The alleles are sorted using the same tree as in panel a. The red lines indicate an abundance of 8.0%.

a

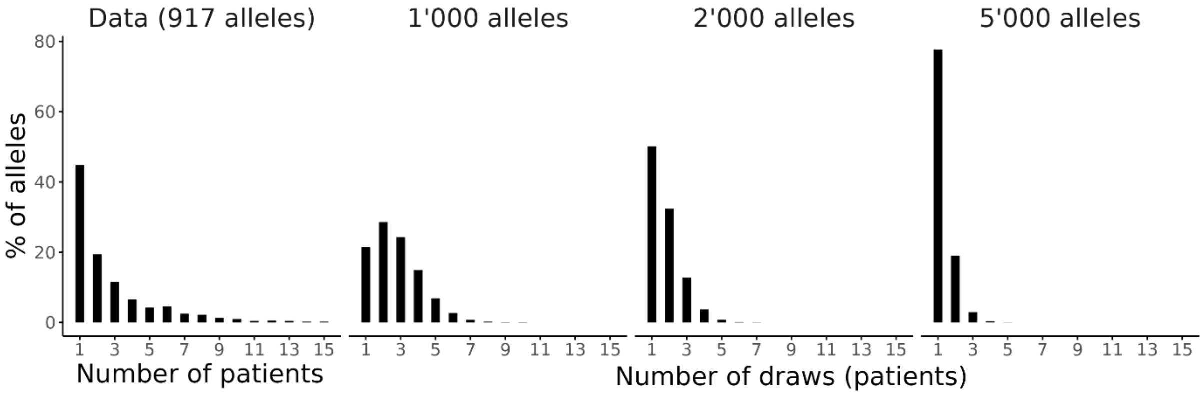

b

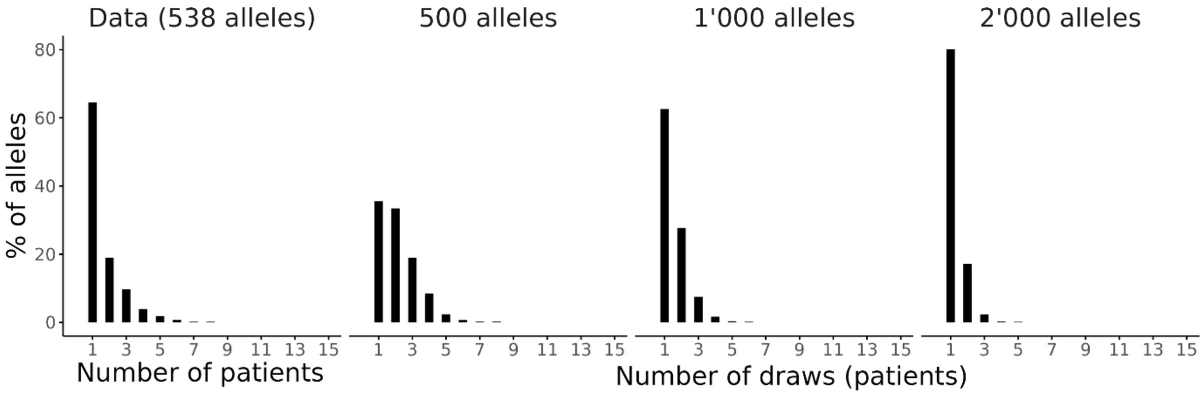

Supplementary Figure 5. **Comparison of the distribution of the alleles observed in the genomic (a) and expressed (b) repertoires of the 24 patients with those obtained by simulating reservoirs of alleles of increasing size (see text).** The software R was used. Data are provided in the Source Data file.

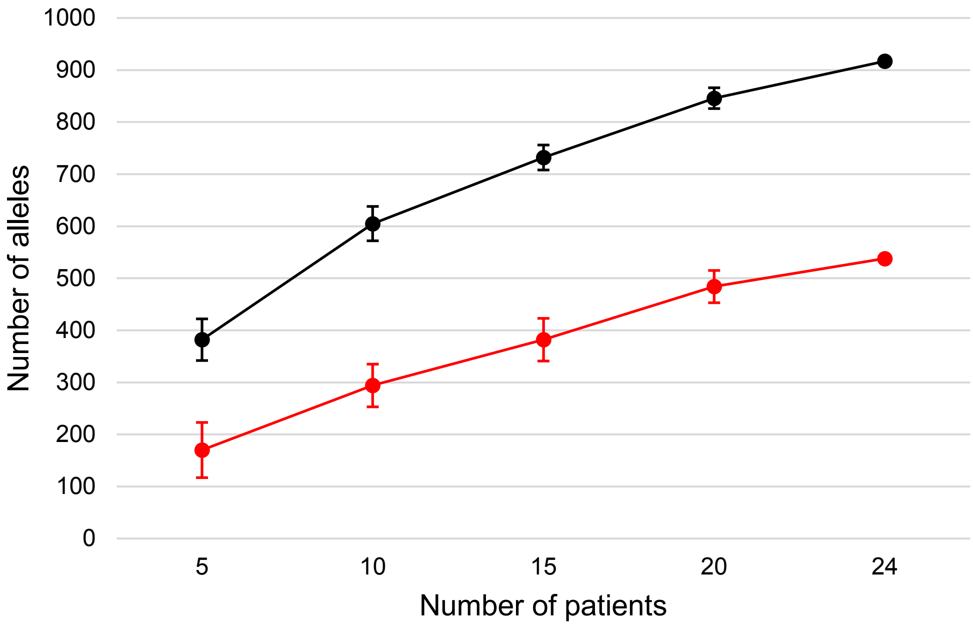

Supplementary Figure 6. Number of alleles observed in the genomic (black) and expressed (red) repertoires in function of the simulated number of patients analysed (see text). SD are shown. The software R was used. Data are provided in the Source Data file.

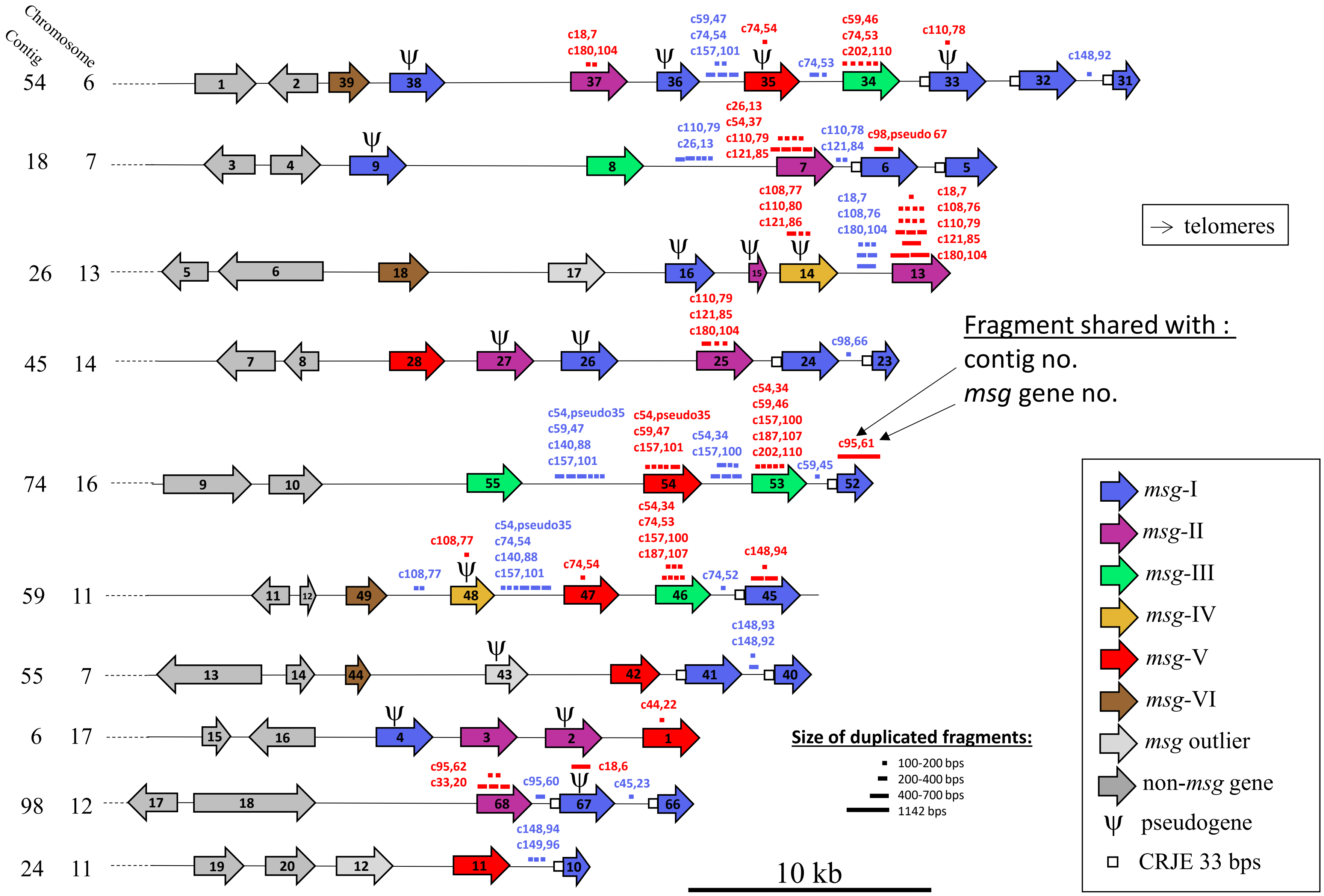

Supplementary Figure 7.

Supplementary Figure 7. **Ten representative subtelomeres (*i.e.* contigs, see Methods) which genes, pseudogenes, and intergenic spaces were used as queries against the whole genome assembled from a single *P. jirovecii* strain using PacBio sequencing**. Adapted from Fig. 1 of reference [1] (reproduced under Creative Commons Attribution 4.0 International license). The symbols represent the size of the duplicated fragments identified within genes and pseudogenes (red symbols), or intergenic spaces (blue symbols). The position of the symbols is not precise. The positions of the duplicated fragments within the query and the subtelomeres are given in Supplementary Data 3 and 4. Adapted from Fig. 3 of Schmid-Siegert, E. et al. Mechanisms of surface antigenic variation in the human pathogenic fungus *Pneumocystis jirovecii*. MBio 8, 1–17 (2017), and reproduced under Creative Commons Attribution 4.0 International license (https://creativecommons.org/licenses/by/4.0).

a

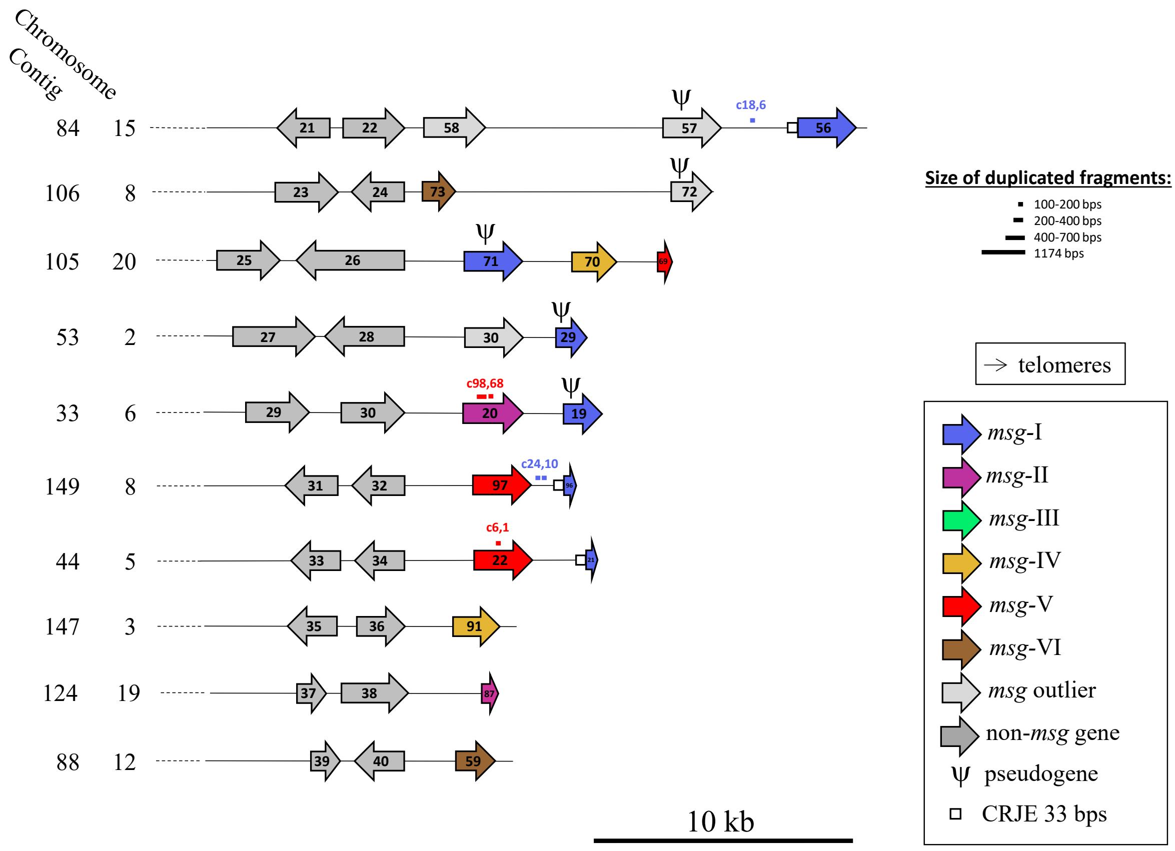

Supplementary Figure 8.

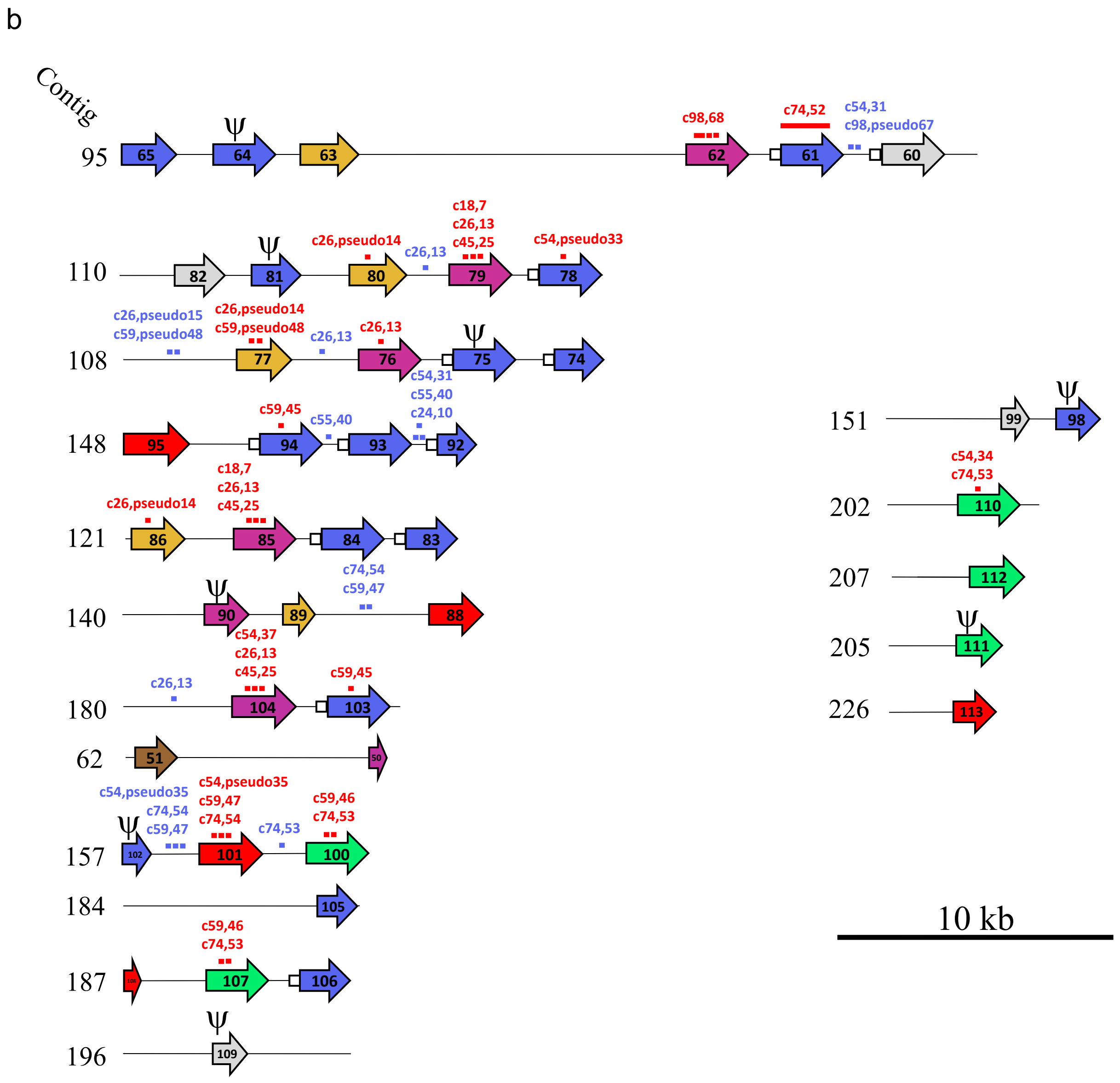

Supplementary Figure 8. **Supplementary subtelomeres (*i.e.* contigs, see methods) from the same single strain as the 10 representative ones shown Supplementary Fig. 7**. The symbols represent the size of the duplicated fragments identified within genes and pseudogenes (red symbols), or intergenic spaces (blue symbols). Their positions within the query and the subtelomeres are given in Supplementary Data 3 and 4. Adapted from Supplementary Fig. 6 of Schmid-Siegert, E. et al. Mechanisms of surface antigenic variation in the human pathogenic fungus *Pneumocystis jirovecii*. MBio 8, 1–17 (2017), and reproduced under Creative Commons

Attribution 4.0 International license (https://creativecommons.org/licenses/by/4.0).

a. 10 subtelomeres harboring genomic genes (in gray) that allowed their attribution to a specific chromosome from reference [14].

b. 17 subtelomeres not harboring genomic genes, preventing their attribution to a chromosome.

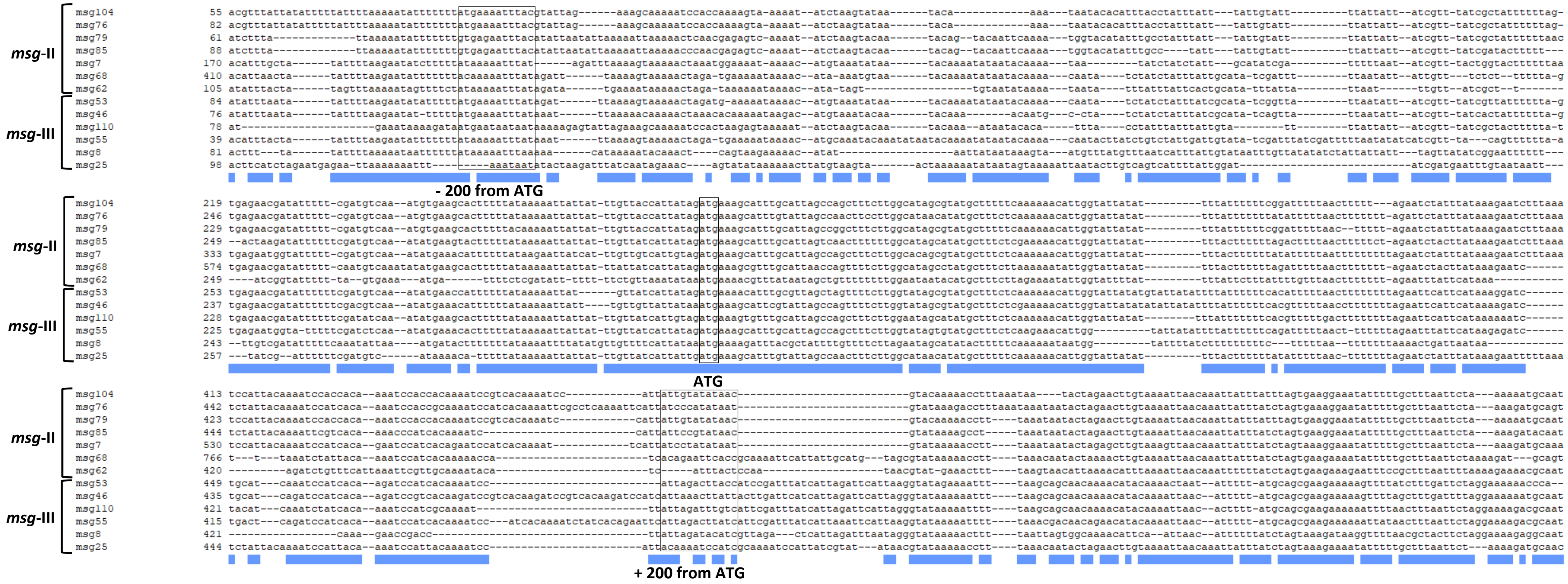

Supplementary Figure 9. Alignments of the region surrounding the ATG of seven *msg*-II and six *msg*-III genes presenting significant similarity in the 200 bps upstream of their CDS. The blue bars underneath indicate areas of significant similarity. Such areas are particularly long around the start codon ATG of the CDS. The ATG start codons and the regions encompassing the – and + 200 bps positions relatively to the ATG of sequences are boxed.

.

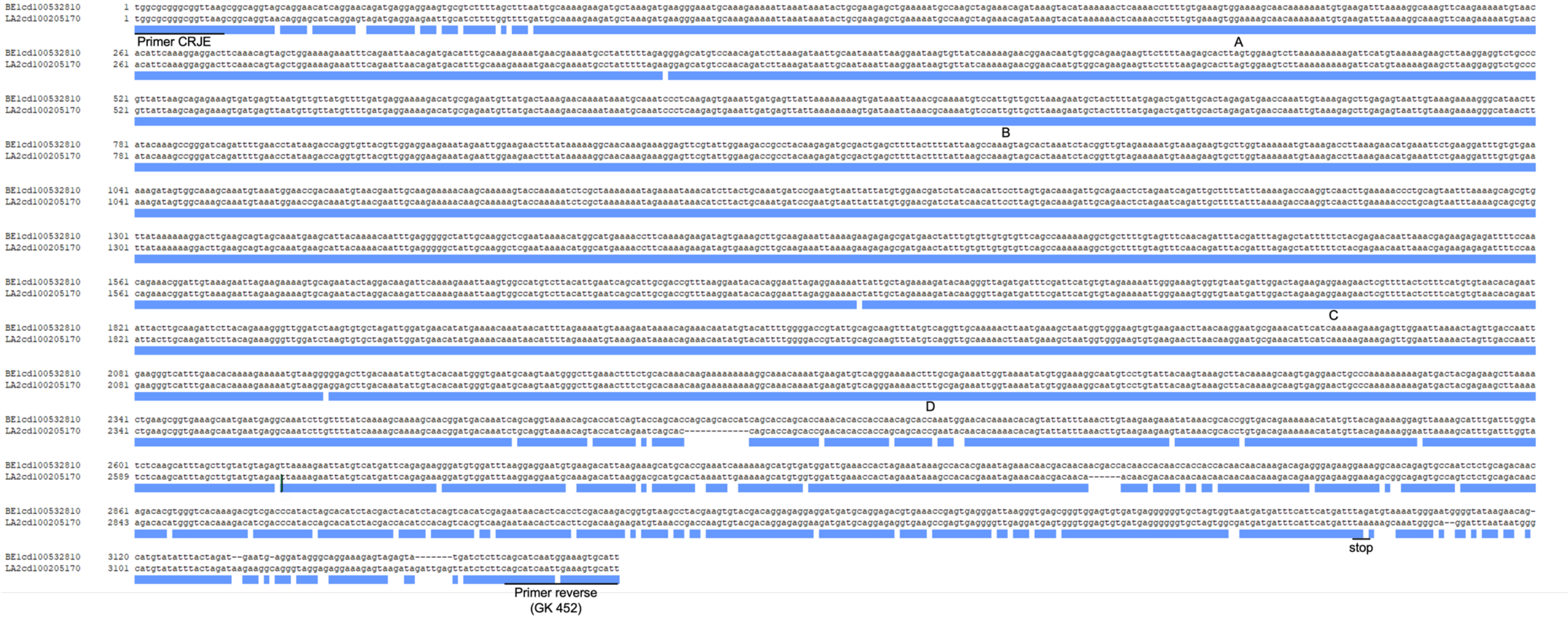

Supplementary Figure 10. Alignment of two mosaic *msg*-I genes identified in this study (the names of the alleles are given on the left). The blue bars indicate areas of full identity. The four fragments of ≥ 100 bps shared are indicated (A to D, respectively of 284, 1335, 420, and 294 bps). Fragment C is shown in red within Figure 6. Clone Manager 9 Professional Edition software version 9.51 (Sci Ed Software LLC) and the alignment format “similarity summary bars” were used.

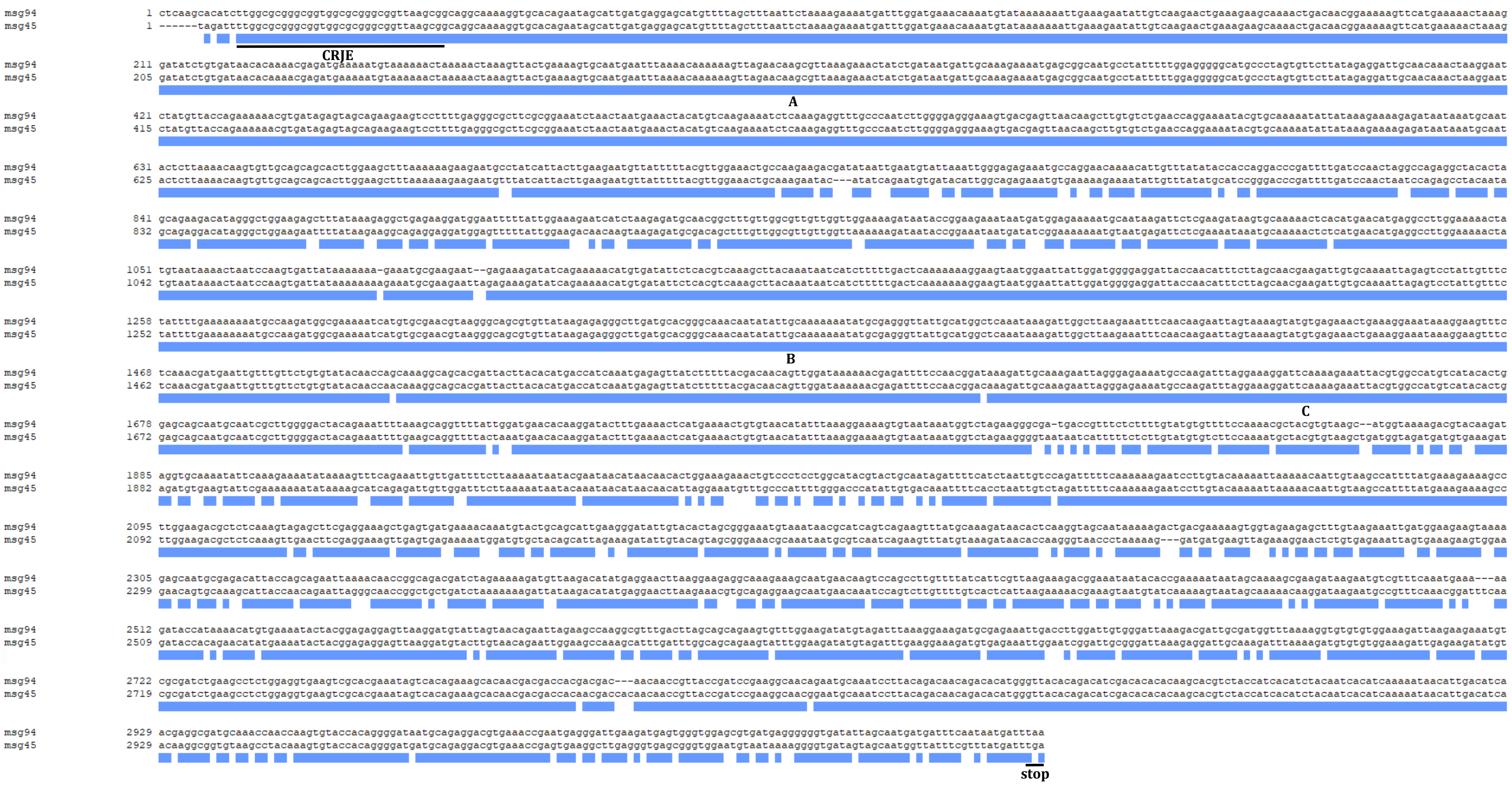

Supplementary Figure 11. Alignment of the mosaic *msg*-I genes no. 45 and 94. The blue bars indicate areas of full identity. The three fragments of $\geq$ 100 bps shared are numbered (respectively 670, 427, and 118 bps). Clone Manager 9 Professional Edition software version 9.51 (Sci Ed Software LLC) and the alignment format “similarity summary bars” were used.

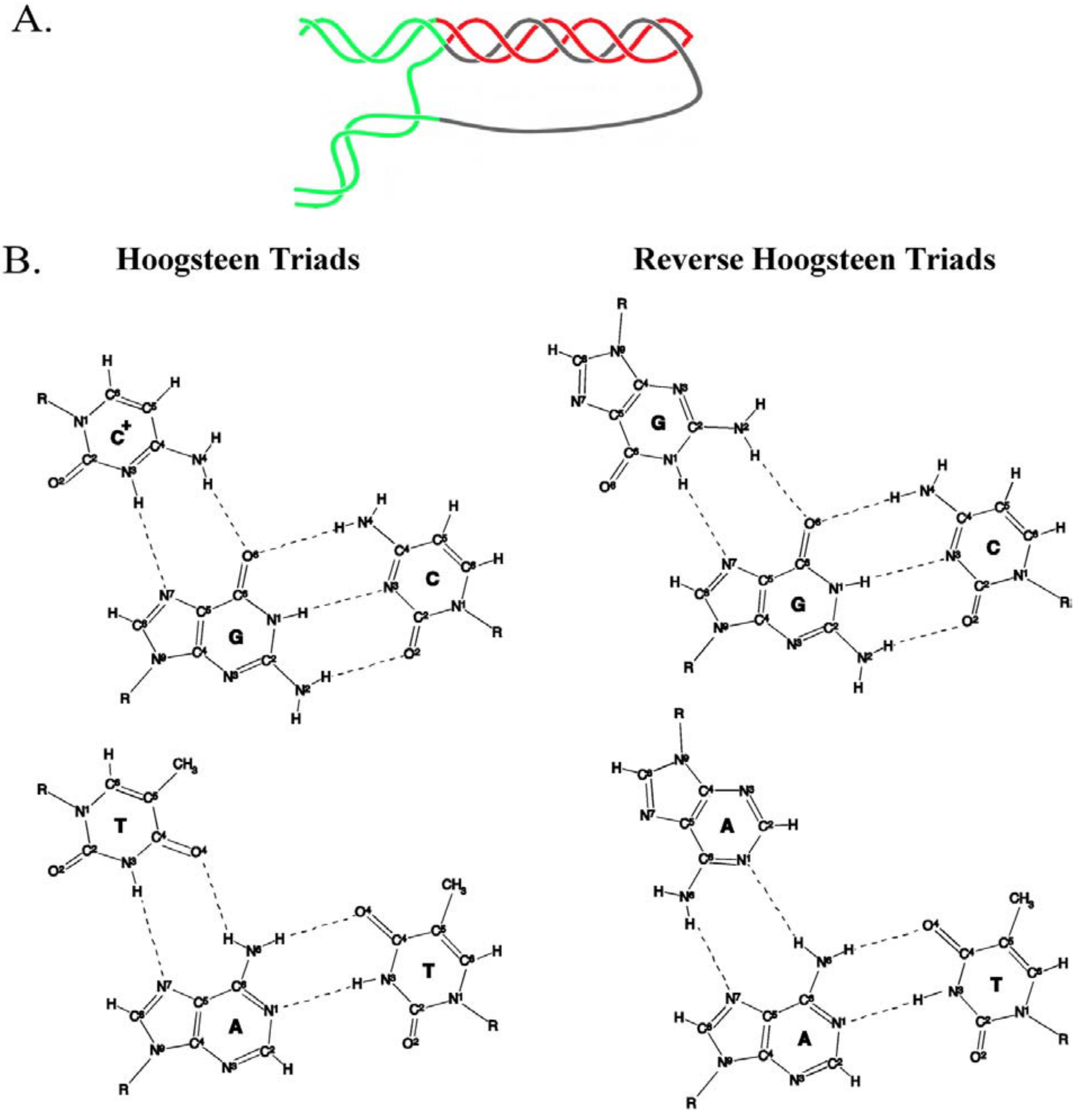

Supplementary Figure 12.

C.

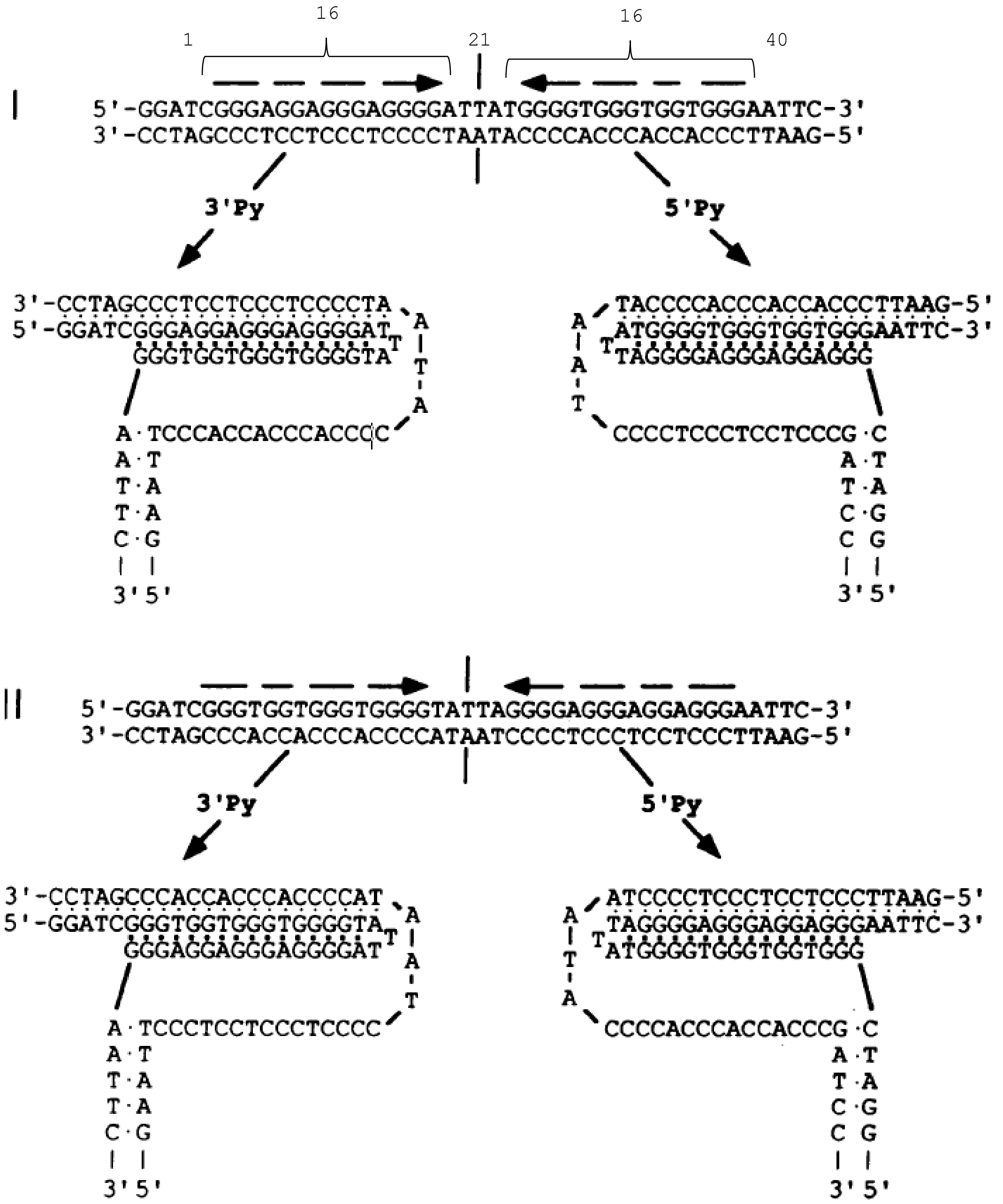

Supplementary Figure 12.

Supplementary Figure 12. Features of H-DNA and *H-DNA triplexes. A. and B.: Figure 3 and its legend from reference [15]. C.: Figure 1 and its legends from reference [16], with added numbering. All three panels are reproduced with permission from corresponding author and copyright holder S. M. Mirkin.

A. The structure of an intramolecular triplex. The two complementary strands of a homopurine-homopyrimidine repeat are colored in red and gray, while flanking DNA is colored green. The structure is called H-y when the red strand is homopyrimidine, and H-r ~~if~~ when it is homopurine. One can see that the red and gray strands in this structure are not linked, *i.e.* formation of H-DNA is topologically equivalent to an unwinding of the entire homopurine-homopyrimidine repeat.

B. H-y form is built from TA*T and CG*C^{+} triads, in which pyrimidines in the third strand form Hoogsteen hydrogen bonds with the purines of the duplex. H-r form and is built of CG*G and TA*A triads, where purines from the third strand form reverse Hoogsteen hydrogen bonds with the purines in the duplex.

C. Intramolecular triplexes consisting of GG*C and TA*T triads. In both sequences I and II, GC base pairs are arranged as mirror images (shown by arrows; the pseudosymmetry axis is shown by a vertical line), whereas AT base pairs are arranged as inverted repeats. Points, Watson-Crick hydrogen bonds; squares, Hoogsteen hydrogen bonds.

Group 1

P. jirovecii
Human

```
    11            11
1            14            27    33
5′ TTGGCGCGGGCGGTGGCGCGGGCGGTTAAGCGG 3′      73% (16/22)
    L  A  R  A  V  A  R  A  V  K  R
```

P. macacae
Rhesus macaque

```
    11            11
1            14                  33
5′ TTGGCGCGGGGGGCGGCGCGGGCGGTCAAGAGG 3′      77% (17/22)
    L  A  R  G  A  A  R  A  V  K  R
```

P. oryctolagi
European rabbit

```
                  17                 11
1                           29                 48
5′ CCCCAGAATTTGGCGCGGGCGGCGGCAGTGCCCCGGGCGGTGAAGAGG 3′      64%(18/28)
    P  Q  N  L  A  R  A  A  A  V  P  R  A  V  K  R
```

Group 2

P. carinii
Norway or brown rat

```
       14         11
1               17 19          32      41
5′ TTGTCCAAGAGGTGGCAATGGCACGGCCGGTTAAGAGG 3′      64% (16/25)
      V  Q  E  V  A  M  A  R  P  V  K  R
```

P. murina
House mouse

```
          19                 11
1                     23                   44
5′ AAGTCGCCCAGAAAGAGGCGGCGATGGCACAGCCGGTTAAGAGG 3′      67% (20/30)
      V  A  Q  K  E  A  A  M  A  Q  P  V  K  R
```

P. sp. "fluvescens"
Chestnut white-bellied rat

```
                   19                 13
1                            30                   50
5′ TTGAGGAAGTCGCCCAGAAAGAGGCGGCGATGGCACAGCCGGTCAAGAGG 3′      63% (20/32)
      E  E  V  A  Q  K  E  A  A  M  A  Q  P  V  K  R
```

P. sp. "muelleri"
Müller's giant Sunda rat

```
                   19                  13
1                              31                   51
5′ TGGCTAGAAGTCGCGCAGAAAGAGGCGGCGATGGCACAGCCGGTCAAGAGG 3′      63% (20/32)
    W  L  E  V  A  Q  K  E  A  A  M  A  Q  P  V  K  R
```

Supplementary Figure 13a.

Group 3

P. wakefieldiae
Norway or brown rat

```
                                       20
   1                    ____________________________           51
5′ TGGTTTGAGCATGGGTTTGACTTGGGAGAAGCGGCACAGCCGGTCAAGAGG 3′    80% (16/20)
     W  F  E  H  G  F  D  L  G  E  A  A  Q  P  V  K  R
```

P.* sp. *“tanezumi”
Asian house rat

```
                                    27
   1                 ______________________________________     52
5′ TTGGTTTGAGCATGGGTTTGACTTGGAAGAAGCGGCACAGCCGGTCAAGAGG 3′    63% (17/27)
      W  F  E  H  G  F  D  L  E  E  A  A  Q  P  V  K  R
```

P.* sp. *“exulans”
Polynesian rat

```
              16
   1  ____________________      30
5′ TTGGAAGAAGCGGCACAGCCGGTCAAGAGG 3′    75% (15/20)
     L  E  E  A  A  Q  P  V  K  R
```

Outlier

P. canis
Dog

```
         16              8
   ______________  __________
   1            14                  33
5′ GGCAGGGAGGGGGTGCAGATGCTTAAGAAGAGG 3′    79% (19/24)
     G  R  E  G  V  Q  M  L  K  K  R
```

Supplementary Figure 13a.

```
P canis            1 ------------------------GGCAGGGAGGGGGTGCAGATGCTTAAG------AAGAGG   Outlier
P exulans          1 ---------------------------TTGGAAGAAGCGGC------ACAGCCGGTCAAGAGG
P tanezumi         1 ------TGGTTTGAGCATGGGTTTGACTTGGAAGAAGCGGC------ACAGCCGGTCAAGAGG   Group 3
P wakefieldiae     1 ------TGGTTTGAGCATGGGTTTGACTTGGGAGAAGCGGC------ACAGCCGGTCAAGAGG
P carinii          1 ----------------------TTGTCCA---AGAGGTGGCAATGGCACGGCCGGTTAAGAGG
P muelleri         1 ---------TGGCTAGA---AGTCGCGCAGAAAGAGGCGGCGATGGCACAGCCGGTCAAGAGG   Group 2
P fulvescens       1 ---------------GAGGAAGTCGCCCAGAAAGAGGCGGCGATGGCACAGCCGGTCAAGAGG
P murina           1 GACAAGCGATGGGTAGAGGAAGTCGCCCAGAAAGAGGCGGCGATGGCACAGCCGGTTAAGAGG
P macacae          1 ---------T------------TGGCGCGG---GGGGCGGCG------CGGGCGGTCAAGAGG
P jirovecii        1 ---------T------------TGGCGCGG---GCGGTGGCG------CGGGCGGTTAAGCGG   Group 1
P oryctolagi       1 CCCCAGAATT------------TGGCGCGG---GCGGCGGCAGTGCCCCGGGCGGTGAAGAGG
```

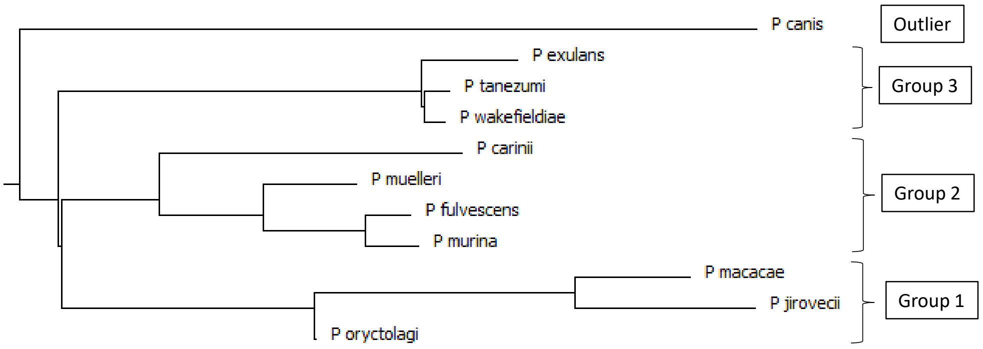


```
P canis                 1 ----------GREGVQMLKKR   Outlier
P sp "exulans"          1 ----------LEEA-AQPVKR
P sp "tanezumi"         1 ---WFEHGFDLEEA-AQPVKR   Group 3
P wakefieldiae          1 ---WFEHGFDLGEA-AQPVKR
P sp "fulvescens    "   1 -----EEVAQKEAAMAQPVKR
P murina                1 DKRWVEEVAQKEAAMAQPVKR   Group 2
P sp "muelleri"         1 ----WLEVAQKEAAMAQPVKR
P carinii               1 ---------VQEVAMARPVKR
P macacae               1 -----LARG---AA--RAVKR
P jirovecii             1 -----LARA---VA--RAVKR   Group 1
P oryctolagi            1 --PQNLARA---AAVPRAVKR
```

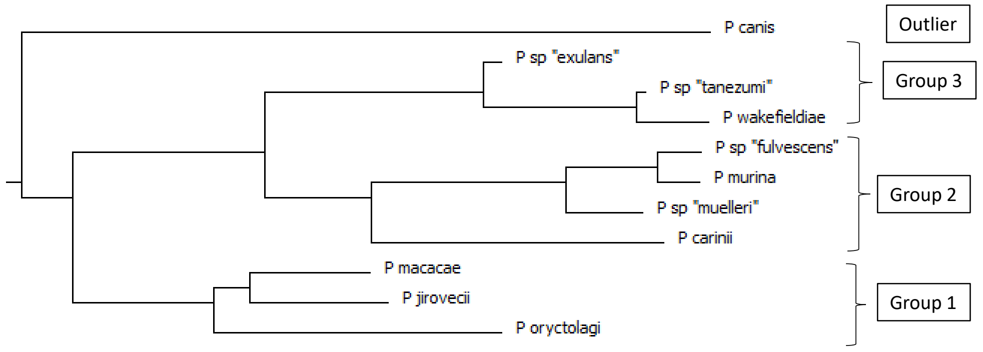

Supplementary Figure 13b.

Supplementary Figure 13. Features of the CRJE of 11 *Pneumocystis* species that is present at the end of the UCS and at the beginning of each *msg*-I gene. The specific mammalian host is given under the *Pneumocystis* species name. The CRJE sequences are those present in Figure 4 of Ma et al[20]. Their NCBI GenBank accession numbers are: *P. jirovecii* (T551_00002), *Pneumocystis sp. "macacae"* (MN509821), *Pneumocystis oryctolagi* (MN507527), *P. carinii* (T552_04149), *P. murina* (PNEG_04309), *Pneumocystis sp. "fulvescens"* (MN509819), *Pneumocystis sp. "muelleri"* (MN509817), *Pneumocystis wakefieldiae* (AF164562), *Pneumocystis sp. "tanezumi"* (MN509820), *Pneumocystis sp. "exulans"* (MN509818), and *Pneumocystis canis* (MN509823). In absence of the sequences of several *msg*-I genes, the 5' extremity of each CRJE is approximate for all species except *P. jirovecii*, *P. carinii*, *P. murina* and *P. oryctolagi*. The sequence of the CRJE of *P. murina* shown is only the 44 out of 132 bps that are left of the Kexin site[24].

(a) The strand of the CRJE shown is enriched in purines and encodes the peptide given underneath that is part of the Msg-I protein. For group 1, the mirror repeat is symbolized by the convergent arrows and the imperfect positions are underlined. The recognition site of the Kexin KR is underlined as well as its encoding codons. Cytosines at imperfect positions of the mirror repeat lead to all R residues present in the peptides in addition to that present in the site KR. The percentage of purines within the enriched stretch(es) identified arbitrarily by arrows or lines above the nucleotide sequence are given on the right.

(b) The alignments of the DNA sequences and encoded peptides of the 11 CRJEs were obtained using Clone Manager 9 Professional Edition software version 9.51 (Sci Ed Software LLC) and the "similarity format" with areas of high matches colored in blue. The linear scoring matrix was used for the corresponding trees shown.

Supplementary References

1. Schmid-Siegert, E. *et al.* Mechanisms of surface antigenic variation in the human pathogenic fungus *Pneumocystis jirovecii*. *MBio* **8**, 1–17 (2017).
2. Morgan, M. BiocManager: Access the Bioconductor project package repository. (2021).
3. Pagès, H., Aboyoun, P., Gentleman, R. & DebRoy, S. Biostrings: Efficient manipulation of biological strings. (2019).
4. Wright, Erik, S. Using DECIPHER v2.0 to analyze big biological sequence data in R. *R J.* **8**, 352 (2016).
5. Galili, T. dendextend: an R package for visualizing, adjusting, and comparing trees of hierarchical clustering. (2015) doi:10.1093/bioinformatics/btv428.
6. Wickham, H. *ggplot2: Elegant graphics for data analysis*. (Springer-Verlag New York, 2016).
7. Warnes, G. R. *et al.* gplots: Various R programming tools for plotting data. (2020).
8. Auguie, B. gridExtra: Miscellaneous functions for ‘grid’ graphics. (2017).
9. Schulz, A. pBrackets: Plot brackets. (2021).
10. Lemon, J. Plotrix: a package in the red light district of R. *R-News* **6**, 8–12 (2006).
11. Wickham, H. *Reshaping data with the {reshape} package*. *Journal of Statistical Software* vol. 21 (2007).
12. Charif, D. & Lobry, J. R. Seqin{R} 1.0-2: a contributed package to the {R} project for statistical computing devoted to biological sequences retrieval and analysis. in *Structural approaches to sequence evolution: Molecules, networks, populations* (eds. Bastolla, U., Porto, M., Roman, H. E. & Vendruscolo, M.)

207–232 (Springer Verlag, 2007).

13. Wickham, H. *stringr: Simple, consistent wrappers for common string operations*. *R package version* (2015).

14. Ma, L. *et al.* Genome analysis of three *Pneumocystis* species reveals adaptation mechanisms to life exclusively in mammalian hosts. *Nat. Commun.* **7**, (2016).

15. Mirkin, S. M. Discovery of alternative DNA structures: a heroic decade (1979-1989). *Front. Biosci.* **13**, 1064 (2008).

16. Dayn, A., Samadashwily, G. M. & Mirkin, S. M. Intramolecular DNA triplexes: unusual sequence requirements and influence on DNA polymerization. *Proc. Natl. Acad. Sci.* **89**, 11406–11410 (1992).

17. Grindley, N. D. F., Whiteson, K. L. & Rice, P. A. Mechanisms of site-specific recombination. Ann. Rev. Biochem. **75**, 567–695 (2006).

18. Hauser, P.M., Francioli, P., Bille, J., Telenti, A., Blanc, D.S. Typing of *Pneumocystis carinii* f. sp. *hominis* by single-strand conformation polymorphism of four genomic regions. J. Clin. Microbiol. **35**, 3086-3091 (1997).

19. Gianella, S., Haeberli, L., Joos, B., Ledergerber, B., Wüthrich, R.P., Weber, R., Kuster, H., Hauser, P.M., Fehr, T. & Mueller, N.J. Molecular evidence of interhuman transmission in an outbreak of *Pneumocystis jirovecii* pneumonia among renal transplant recipients. Transpl. Infect. Dis.. **12**, 1-10 (2010).

20. Ma, L. et al. Diversity and complexity of the large surface protein family in the compacted genomes of multiple *Pneumocystis* species. MBio 11, 1–20 (2020).

21. Cissé, O.H. et al. Genomic insights into the host specific adaptation of the *Pneumocystis* genus. Com. Biol. **4**, 305 (2021).

22. Mirkin, S. M. & Frank-Kamenetskii, M. D. H-DNA and related structures. Annu. Rev. Biophys. Biomol. Struct. 23, 541–576 (1994).

23. Xue, T. *et al.* Genotyping of *Pneumocystis jirovecii* by use of a new simplified nomenclature system based on the internal transcribed spacer regions and 5.8S rRNA gene of the rRNA operon. *J. Clin. Microbiol.* **57**, e02012-18 (2019).

24. Keely, S. P., Linke, M. J., Cushion, M. T. & Stringer, J. R. *Pneumocystis murina* MSG gene family and the structure of the locus associated with its transcription. Fungal Genet. Biol. **44**, 905–919 (2007).
