# Supplementary material for: Fungal antigenic variation using mosaicism and reassortment of subtelomeric genes’ repertoires
Source: Nat Commun. 2023 Nov 2;14:7026. doi: 10.1038/s41467-023-42685-6 (PMC10622565; doi:10.1038/s41467-023-42685-6)
Supplement: Supplementary file 3 — Description of Additional Supplementary Files [file 41467_2023_42685_MOESM3_ESM.pdf]

Description of Additional Supplementary Files

File Name: Supplementary Data 1
Description: Similarities to the 200 bps up- and downstream of 20 representative *msg* CDs within the 10 representative subtelomeres shown in Supplementary Fig. 7. The 200 bps up- or downstream of each gene was used as query in BLASTn analyses against the *P. jirovecii* PacBio genome assembly of reference 1. All significant hits with > 40% coverage of the query are listed (the 6 hits in red were localized several thousand bps from *msg* genes, not immediately up- or downstream as the other hits).

File Name: Supplementary Data 2
Description: Identities between genes and between the 200 bps sequences localized immediately up- or downstream of representative *msg* genes of each family. The identities between the 200 bps sequence located immediately up- or downstream of the *msg* CDS and all their BLASTn hits shown in Table 2 and Supplementary Data 1 are given. The *msg* gene numbers are those used in Supplementary Fig. 7. Note that partial msg of family IV no. 89 was not analysed because it could not be aligned properly.

File Name: Supplementary Data 3
Description: Duplicated fragments > 100 bps shared by the genes and pseudogenes of the 10 representative subtelomeres shown in Supplementary Fig. 7. The genes and pseudogenes were used as query in BLASTn analyses against the *P. jirovecii* PacBio genome assembly of reference 1. The fragments shared by the query and each hit were retrieved by inspection of the alignments (the 20 fragments in red were detected twice in reciprocal BLAST analyses but counted only once in Table 2).

File Name: Supplementary Data 4
Description: Duplicated fragments > 100 bps shared by the upstream intergenic spaces of the 10 representative subtelomeres shown in Supplementary Fig. 7. The upstream intergenic spaces were used as query in BLASTn analyses against the *P. jirovecii* PacBio genome assembly of reference 1. The fragments shared by the query and each hit were retrieved by inspection of the alignments (the 28 fragments in red were detected twice in reciprocal BLAST analyses but counted only once in Supplementary Data 1).

File Name: Supplementary Data 5
Description: Relative abundance of the *msg*-I alleles in the patients (in %).

File Name: Supplementary Data 6
Description: Contains tables 1 and 2. Table 1 is the contingency table of the 917 and 538 alleles of the genomic and 24 expressed msg-I repertoires among the cities and patients. Table 2 shows the homologies to the 200 bps sequences localized immediately up- or downstream of 20 representative msg CDSs within P. jirovecii subtelomeres from a single strain.
