# Supplementary material for: Fungal antigenic variation using mosaicism and reassortment of subtelomeric genes’ repertoires
Source: Nat Commun. 2023 Nov 2;14:7026. doi: 10.1038/s41467-023-42685-6 (PMC10622565; doi:10.1038/s41467-023-42685-6)
Supplement: Supplementary file 7 — Reporting Summary [file 41467_2023_42685_MOESM7_ESM.pdf]

nature portfolio

Corresponding author(s): Hauser Philippe

Last updated by author(s): 21/9/23

Reporting Summary

Nature Portfolio wishes to improve the reproducibility of the work that we publish. This form provides structure for consistency and transparency in reporting. For further information on Nature Portfolio policies, see our Editorial Policies and the Editorial Policy Checklist.

Statistics

For all statistical analyses, confirm that the following items are present in the figure legend, table legend, main text, or Methods section.

n/a	Confirmed	
☐	☒	The exact sample size (*n*) for each experimental group/condition, given as a discrete number and unit of measurement
☒	☐	A statement on whether measurements were taken from distinct samples or whether the same sample was measured repeatedly
☐	☒	The statistical test(s) used AND whether they are one- or two-sided<br>*Only common tests should be described solely by name; describe more complex techniques in the Methods section.*
☒	☐	A description of all covariates tested
☒	☐	A description of any assumptions or corrections, such as tests of normality and adjustment for multiple comparisons
☐	☒	A full description of the statistical parameters including central tendency (e.g. means) or other basic estimates (e.g. regression coefficient) AND variation (e.g. standard deviation) or associated estimates of uncertainty (e.g. confidence intervals)
☐	☒	For null hypothesis testing, the test statistic (e.g. *F*, *t*, *r*) with confidence intervals, effect sizes, degrees of freedom and *P* value noted<br>*Give P values as exact values whenever suitable.*
☒	☐	For Bayesian analysis, information on the choice of priors and Markov chain Monte Carlo settings
☒	☐	For hierarchical and complex designs, identification of the appropriate level for tests and full reporting of outcomes
☐	☒	Estimates of effect sizes (e.g. Cohen's *d*, Pearson's *r*), indicating how they were calculated

Our web collection on statistics for biologists contains articles on many of the points above.

Software and code

Policy information about availability of computer code

Data collection	n/a (no software used for data collection)

Data analysis

Softwares used for allele identification and quantification (methods section): hmmer, swarm (version 3.1.0), 3. cd-hit (version 4.8.1).

Softwares used for analyses of DNA sequences similiraties: NCBI BLAST suite (https://blast.ncbi.nlm.nih.gov/Blast.cgi).

R packages used with the R version 4.1.0 (2021-05-18) (Table S2):

BiocManager version 1.30.16 access bioconductor package repository,
biostrings version 2.62.0 manipulation of biological strings,
DECIPHER version 2.22.0 manage biological sequences,
dendextend version 1.15.2 dendogram manipulation,
ggplot version 2 3.3.5 figures and plots,
gplots 3.1.1 create heatmaps,
gridExtra 2.3 arrange plots,
pBrackets 1.0.1 bracket elements in plot,
plotrix 3.8-2 plot options,
reshape2 1.4.4 reshape data,
seqinr 4.2-8 manipulation of sequences,
stringr 1.4.0 string operations.

For manuscripts utilizing custom algorithms or software that are central to the research but not yet described in published literature, software must be made available to editors and reviewers. We strongly encourage code deposition in a community repository (e.g. GitHub). See the Nature Portfolio guidelines for submitting code & software for further information.

Data

Policy information about availability of data

All manuscripts must include a data availability statement. This statement should provide the following information, where applicable:

- Accession codes, unique identifiers, or web links for publicly available datasets
- A description of any restrictions on data availability
- For clinical datasets or third party data, please ensure that the statement adheres to our policy

Data availability statement
The PacBio CCS raw reads generated in this study (accession nos.) have been deposited in the NCBI Sequence Read Archive linked under accession code SRP434110 [https://trace.ncbi.nlm.nih.gov/Traces/?view=study&acc=SRP434110] ; theSRR24284242 to SRR24284301 (msg-I) and SSR25739987 to SRR25740015 (ITS1-5.8S-ITS2) in BioProject accession no. PRJNA936793 and BioSample accession no. SAMN33368625. The sequences obtained in this study have been deposited in Genbank (1007 new msg-I alleles: accession nos. OR489167 to OR490173; 15 new ITS1-5.8S-ITS2 alleles: OR475686 to OR475700 [https://www.ncbi.nlm.nih.gov/nuccore/OR475686.1/ to https://www.ncbi.nlm.nih.gov/nuccore/OR475700.1/]). A table including the relative abundance of each msg-I allele identified in each patient are is provided as a Supplementary Data 5 . Source data are provided with this paper.

Human research participants

Policy information about studies involving human research participants and Sex and Gender in Research.

Reporting on sex and gender	Not used.
Population characteristics	No such data (only city provenance, underlying disease, and year of sample collection are know for the 24 patients)
Recruitment	Randomly chosen patients with Pneumocystis pneumonia with available clinical sample. The only criterion was availability of the sample (such samples are leftovers and difficult to obtain). Such selection had no impact on the results because a very large diversity of the msg-I genes's repertoires has been observed that is independent of the city provenance.
Ethics oversight	Commission Cantonale d'Éthique de la Recherche sur l'Être Humain, http://www.swissethics.ch. French Ministry of Research and the Agence Régionale de l'Hospitalisation. Seville Hospital review board.

Note that full information on the approval of the study protocol must also be provided in the manuscript.

Field-specific reporting

Please select the one below that is the best fit for your research. If you are not sure, read the appropriate sections before making your selection.

☒ Life sciences ☐ Behavioural & social sciences ☐ Ecological, evolutionary & environmental sciences

For a reference copy of the document with all sections, see nature.com/documents/nr-reporting-summary-flat.pdf

Life sciences study design

All studies must disclose on these points even when the disclosure is negative.

Sample size	24 patients. No calculation of the sample size was performed. The sample size was dictated only by the the maximum number of samples that we could obtain from each city. The results obtained demonstrate that this sample size was sufficient to reach the conclusions that we draw in the manuscript.
Data exclusions	no
Replication	Analysis of 8 samples twice (Supplementary data 1., Table S5, Figure S3).
Randomization	N/A. Randomization could not be performed because the samples positive for Pneumocystis are scarce in the clinical practice and, moreover, the volumes left after routine analyses are difficult to obtain for reasearch.
Blinding	Blinding was applied in the laboratory by coding the samples during the analyses.

Reporting for specific materials, systems and methods

We require information from authors about some types of materials, experimental systems and methods used in many studies. Here, indicate whether each material, system or method listed is relevant to your study. If you are not sure if a list item applies to your research, read the appropriate section before selecting a response.

Materials & experimental systems

n/a	Involved in the study
☒	☐ Antibodies
☒	☐ Eukaryotic cell lines
☒	☐ Palaeontology and archaeology
☒	☐ Animals and other organisms
☒	☐ Clinical data
☒	☐ Dual use research of concern

Methods

n/a	Involved in the study
☒	☐ ChIP-seq
☒	☐ Flow cytometry
☒	☐ MRI-based neuroimaging
